# Supplementary material for: Immunogenicity of an Extended Dose Interval for the Ad26.ZEBOV, MVA-BN-Filo Ebola Vaccine Regimen in Adults and Children in the Democratic Republic of the Congo
Source: Vaccines (Basel). 2024 Jul 23;12(8):828. doi: 10.3390/vaccines12080828 (PMC11359010; doi:10.3390/vaccines12080828)
Supplement: Supplementary file 1 [file vaccines-12-00828-s001.zip › vaccines-3050943-supplementary.pdf]

Immunogenicity of an Extended Dose Interval for the Ad26.ZEBOV, MVA-BN-Filo Ebola Vaccine Regimen in Adults and Children in the Democratic Republic of the Congo

Supplementary Materials

Table of Contents

Supplementary Table S1. .... **2**

Supplementary Table S2. .... **3**

Supplementary Figure S1 .... **4**

Supplementary Table S1. Participant eligibility criteria.

**Inclusion criteria for vaccination**
1. Must provide a written or witnessed (if illiterate) informed consent form indicating that he or she understands the reasons for the study and is willing to participate in the study and be vaccinated. If less than 18 years old, must have a parent or guardian that is able to meet this criterion.<br>2. Must be aged 1 year or older (at enrolment to the main study).<br>3. Must be healthy in the investigator's clinical judgment as assessed on the day of vaccination.<br>4. Must be willing to have a photograph taken.<br>5. Participant must be available and willing to participate for duration of study visits and follow up.
**Exclusion criteria for vaccination**
1. Known history of Ebola virus disease.<br>2. Has received any experimental Ebola vaccine less than one month prior to dose 1 vaccination.<br>3. Known allergy or history of anaphylaxis or other serious adverse reactions to vaccines or vaccine products, egg and egg proteins or gentamicin.<br>4. Presence of acute illness (excluding minor illnesses such as mild diarrhea or mild upper respiratory tract infection) or temperature ≥38.0ºC at the vaccination visit. Participants with such symptoms will be temporarily excluded from vaccination at that time but may be rescheduled for vaccination at a later date if feasible.<br>5. Presence of significant conditions or clinically significant findings at the vaccination visit for which, in the opinion of the investigator, vaccination would not be in the best interest of the participant.
**Contraindications to MVA-BN-Filo (dose 2) vaccination**
A participant would not be given dose 2 vaccination if s/he experienced any of the following events at any time after dose 1 vaccination.
1. Unable to verify dose 1 delivered<br>2. Anaphylaxis considered to be at least possibly related to vaccination with study vaccine; *OR*<br>3. A Serious Adverse Event considered to be at least possibly related to study vaccine; *OR*<br>4. Any other concern that the investigator believes may adversely affect the participant's safety; *OR*<br>5. Laboratory-confirmed EVD before or on day of scheduled dose 2 vaccination.

**Criteria for Postponement of vaccination**
A participant experiencing any of the events or taking any of the concomitant vaccines and medications described below at the vaccination visit may be vaccinated at a later date.
• Acute illness at the time of vaccination (excluding minor illnesses such as diarrhea or mild upper respiratory tract infection); *OR*<br>• Fever (body temperature) ≥38ºC at the time of vaccination; *OR*<br>• Has received immunization with live attenuated vaccines within 30 days; *OR*<br>• Has received immunization with inactivated vaccines within 15 days; *OR*<br>• Has received a standard course of malaria therapy within 3 days.

Supplementary Table S2. Participants who had previously received the rVSV-ZEBOV-GP Ebola vaccine. Two adult participants were excluded from immunogenicity analysis.

Participant ID	Sample	EBOV GP antibody GMC (EU/mL)	Dose 1 and 2 interval (days)
1103984	Pre-dose 2	7429	246
	21 days post-dose 2	60754	
1303326	Pre-dose 2	3442	244
	21 days post-dose 2	16764	

EBOV (Ebola virus); EU (ELISA Unit); GMC (Geometric mean concentration); GP (glycoprotein)

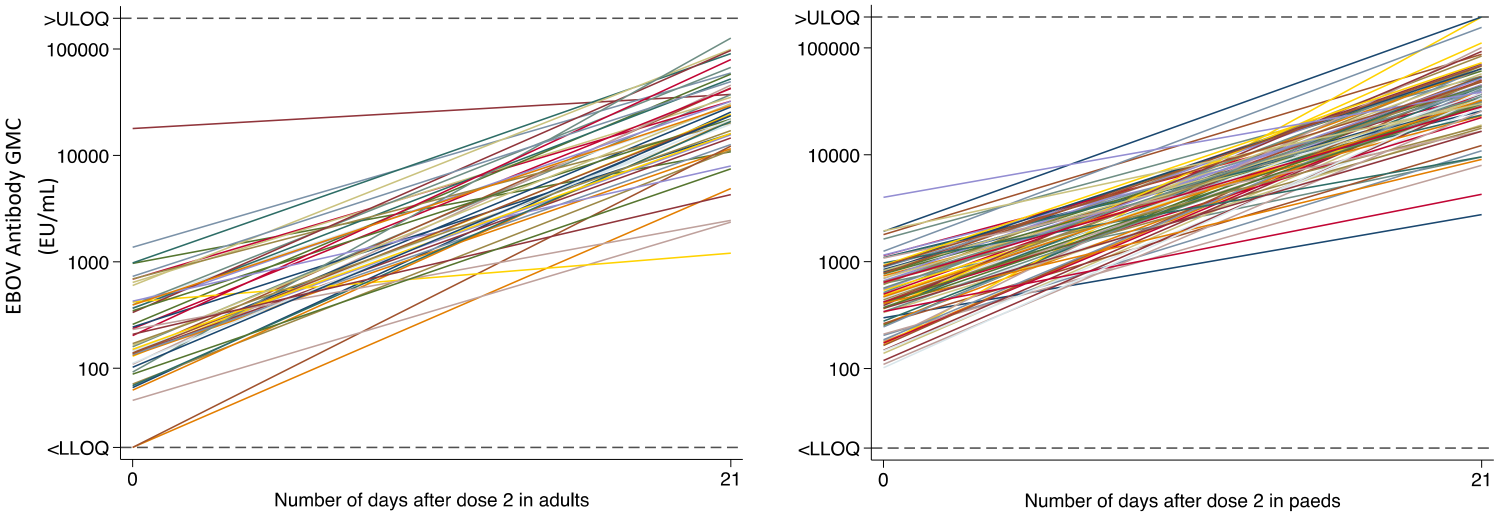

Supplementary Figure S1. Individual trajectories of Ebola virus glycoprotein-specific IgG binding antibody responses over time. Antibody geometric mean concentration (GMC, ELISA Unit (EU)/mL) in adult (18 years or above) and paediatric participants (paeds; 4-17 years). EBOV (Ebola virus); LLOQ (Lower limit of quantification); ULOQ (Upper limit of quantification).
